# Treatment options for women with heavy menstrual bleeding: a protocol for comprehensive systematic review, network meta-analyses and health economic assessment

**DOI:** 10.1136/bmjopen-2024-085292

**Published:** 2025-04-22

**Authors:** Bassel H Al Wattar, Ewelina Rogozińska, Lily Nicholson, David J Fisher, Ekaterina Bordea, Niccola Hutchinson-Pascal, Ngawai Moss, Rachael M Hunter, Khalid Saeed Khan, Davor Jurkovic, Jayne F Tierney, Claire L Vale

**Affiliations:** 1 Obstetrics and Gynaecology, Epsom and Saint Helier Hospital NHS Trust, Carshalton, UK; 2 Clinical Trials Unit, Anglia Ruskin University—Chelmsford Campus, Chelmsford, UK; 3 Meta-analysis Group, MRC Clinical Trials Unit, University College London, London, UK; 4 Comprehensive Clinical Trials Unit, University College London, London, UK; 5 Co-Production and Public Engagement, University College London, London, UK; 6 Patient and Public Representative, University College London, London, UK; 7 Research Department of Primary Care and Population Health, University College London, London, UK; 8 Public Health, University of Granada Faculty of Medicine, Granada, Spain; 9 Gynaecology, University College Hospital, London, UK

**Keywords:** Systematic Review, Health economics, Reproductive medicine, GYNAECOLOGY

## Abstract

**Abstract:**

**Introduction:**

A quarter of women experience heavy periods in their lifetime, often significantly impairing their well-being, productivity and quality of life.

Several treatment options are offered for heavy menstrual bleeding; however, there is limited evidence on the effectiveness, safety and cost of available treatments. We aim to conduct a comprehensive systematic review, network meta-analyses and health economic evaluation to compare all available treatment options while considering the views and treatment preferences of women with heavy menstrual bleeding.

**Methods and analysis:**

We will systematically search electronic databases (MEDLINE, EMBASE, CENTRAL) as well as the grey literature, conference proceedings and trial registries to identify all relevant randomised trials that evaluated any medical or surgical treatment for women with heavy menstrual bleeding regardless of their cause compared with placebo or other active treatments.

We will perform pairwise and network meta-analyses using standard methods. We will report primarily on changes in menstrual blood loss (using Pictorial blood loss assessment chart scores or the Alkaline-Haematin method), quality-of-life measures, safety in addition to other important clinical outcomes.

We will develop a health economic model to evaluate the cost-effectiveness of available treatments within a healthcare perspective using data inputs from the planned meta-analyses. We will calculate the incremental cost per change in alternative outcomes and present the net monetary benefit for a range of cost-effectiveness thresholds for quality-adjusted life-year gained. We will conduct consultations and a discrete choice experiment involving patient representatives to capture the factors influencing women’s decision-making and treatment preferences in real life.

**Ethics and dissemination:**

The project was approved by the UCL Institute for Women’s Health Low-Risk Research Ethics Committee (reference: 004_2023_24) and UCL Research Ethics Committee (ID 16351/003) for the planned patient involvement and qualitative research. We will produce an evidence-based decision aid toolkit and will publish the findings in peer-reviewed journals, as well as lay media outputs to inform health professionals, policymakers and the patient community.

**PROSPERO registration numbers:**

https://doi.org/10.17605/OSF.IO/4MUSF, CRD42023468055, CRD42024519622, CRD42024520558 and CRD42024520634.

**STRENGTHS AND LIMITATIONS OF THIS STUDY**

- The planned analysis will offer the most comprehensive evidence synthesis and health economic evaluation of all available treatment options for the management of heavy periods.
- The project will include various subgroup and sensitivity analyses to explore the varied effectiveness of available treatment options across population subgroups of interest.
- The project will incorporate a discrete choice experiment to quantify women’s preferences for different treatments for heavy periods and will be incorporated into the planned health economic evaluation.
- The project will be limited to the use of aggregate data which may limit the ability to adjust for potential effect modifiers such as age, body mass index and ethnicity.

## Introduction

 Heavy menstrual bleeding (HMB) affects one in four women of reproductive age, leading to significant impairment of their quality of life.^1^ The cause of HMB is often unknown, with more than 50 000 women in England and Wales seeking specialist treatment at secondary gynaecology services in the National Health Service (NHS) annually.^2^ Around 28 000 women eventually require surgery to manage their HMB per year.^3^ HMB chronically affects women who are otherwise healthy at varied life stages (adolescents, pre-pregnancy, perimenopause), adversely impacting their well-being and productivity in society. As such, it is important to consider women’s evolving health needs (eg, need for contraception vs the desire to get pregnant) and their treatment preferences to maximise the benefit and uptake of the varied HMB treatment options.

More than 19 different treatment options are currently offered for HMB in the NHS, including medical options (eg, progestogen-releasing intrauterine systems (IUS), contraceptive pills, danazol, ulipristal acetate, non-steroidal anti-inflammatory drugs, antifibrinolytic agents, gonadotropin-releasing hormone agonists) and surgical options (eg, myomectomy, hysterectomy).^4^

Several systematic reviews and meta-analyses compared HMB treatment to date.^5^,^7^ However, precise and up-to-date evidence to inform decisions regarding treatment selection is limited to head-to-head comparisons of individual treatments^8^ and fails to incorporate women’s treatment preferences.^9 10^ Additionally, several new pharmacological treatments (eg, elagolix and ulipristal acetate) have been introduced into clinical practice, yet their safety and effectiveness compared with existing treatment have not been well evaluated.^11^

With newly introduced treatments, persistent uncertainties and gaps in the existing evidence, there is a need for a clear, comprehensive and succinct evidence synthesis to address this uncertainty while taking into consideration the views and treatment preferences of women with HMB.

## Objectives

Our aim is to perform a comprehensive and up-to-date evidence synthesis on the clinical and cost-effectiveness of all available treatment options for women with HMB and better inform care provision for affected women overall and within specific population subgroups.

## Methods

### Protocol development and registration

This protocol was prospectively registered on Open Science Framework (https://doi.org/10.17605/OSF.IO/4MUSF) in addition to PROSPERO database (CRD42023468055, CRD42024519622, CRD42024520558, CRD42024520634) and is reported in line with the Preferred Reporting Items for Systematic Review and Meta-Analysis Protocols (PRISMA-P) 2015 statement.^12^ The pairwise and network meta-analyses will be conducted according to the Cochrane Collaboration methodology and reported in line with the PRISMA statement for systematic reviews with network meta-analysis.^13^ The project will run between 1 June 2023 and 31 May 2025.

### Evidence synthesis of clinical evidence

**Figure 1:**
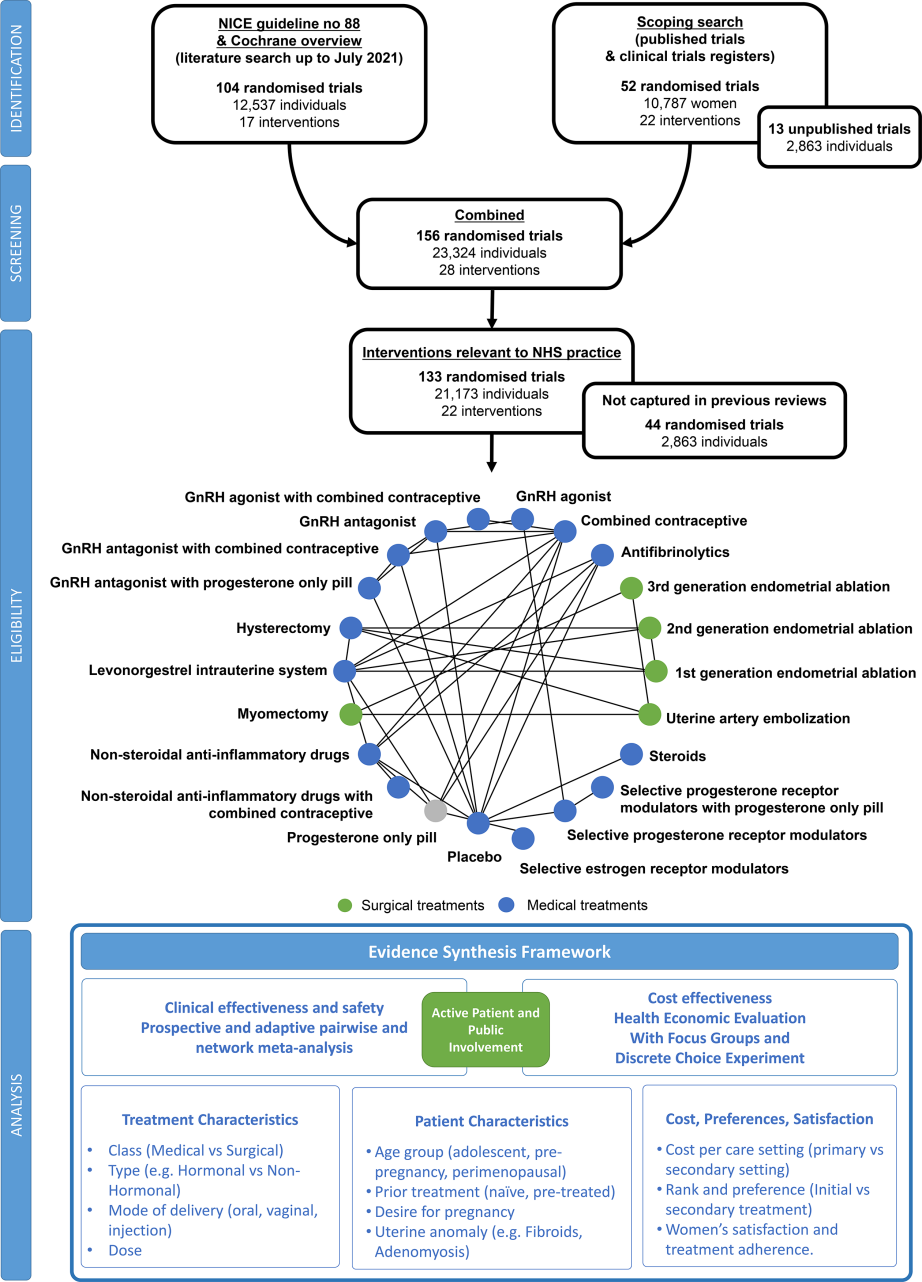
Flow chart of planned systematic review and evidence synthesis evaluating treatment options for heavy menstrual bleeding. GnRH, gonadotropin-releasing hormone; NHS, National Health Service; NICE, National Institute for Health and Care Excellence.

We will conduct a suite of systematic reviews of randomised controlled trials (RCTs) using direct (pairwise) meta-analyses and network meta-analyses to evaluate the effect of different treatment options for HMB compared with placebo, no intervention or other treatment options (figure 1).

### Eligibility criteria

We will include RCTs that involved any individual of reproductive age affected by HMB due to known (eg, uterine fibroid) or unknown causes that compared the following interventions:

- Any hormonal treatment (including combined contraceptives, progesterone-only pills, combined vaginal ring, synthetic steroids, intra-uterine hormone-releasing systems).
- Any pharmacological non-hormonal treatment (including antifibrinolytics or haemostatic agents, anti-inflammatory agents, progesterone receptor modulator agents).
- Surgical treatment options (including open (abdominal), vaginal or laparoscopic hysterectomy, endometrial ablation, hysteroscopic resection of fibroid, myomectomy, uterine artery embolisation).

### Information sources and search strategy

To identify all relevant RCTs, we will perform a multistage systematic search including electronic databases (MEDLINE, EMBASE, CENTRAL) using a multistage strategy combining MeSH terms and keywords using the Boolean operators AND/OR (online supplemental table 1). The search strategy will include terms relating to or describing the population, interventions and study design without any filters or language restrictions. Additionally, we will search international clinical trials registries (Clinicaltrials.gov, and WHO International Clinical Trials Registry Platform) to identify any ongoing and/or recently completed trials and the grey literature searches (eg, proceedings from relevant clinical conferences, including proceedings of the Royal College of Obstetricians and Gynaecologists World Conference); dissertations and theses, Health Technology Appraisals submissions and regulatory submissions) to identify any additional potentially relevant citations in the grey literature. We will manually screen Clinical Practice Guidelines on the management of HMB, relevant Cochrane reviews and bibliographies of relevant articles to identify any missed citations. We will use the summaries available from the previous evidence synthesis, including the National Institute for Health and Care Excellence (NICE) Clinical Practice Guidelines and available Cochrane Reviews on HMB to eliminate duplication of efforts and streamline the extraction of relevant data. Consequently, our literature search will cover a period from 1 January 2019 and will be updated biannually until 6 months before the end of the project. We will exclude quasi and non-randomised studies, reviews and animal studies. Articles in non-English languages will be translated.

### Study selection and data collection

Two independent reviewers will screen relevant citations and full-text articles using the COVIDENCE software (COVIDENCE, Melbourne, Australia). All retrieved trials will be reviewed against our eligibility criteria, recording the reasons for any exclusion. Two reviewers will independently extract data from all eligible studies on the aspects of the trial design, interventions, characteristics of participants at the point of randomisation and outcomes (overall and within prespecified patient subgroups, where available), using a bespoke data extraction tool, similar to those used in previous studies.^14^

We will adopt a collaborative approach to evidence synthesis^14 15^ by maintaining open communication with trial investigators to ensure that data on all outcomes and subgroups of importance are collected for each trial even when reported and will facilitate thorough data checking. Key study information (participants’ characteristics, interventions and outcomes) from each study contributing to the quantitative evidence synthesis will be summarised in a tabulated format. To avoid duplication of efforts, data extraction from previously published Cochrane reviews will be harvested. We will extract data from articles published after 1 January 2019 that met our inclusion criteria. Where additional data are required, we will review and extract data from all primary studies included in our review.

### Quality assessment

We will assess the risk of bias for each study using the criteria of the Cochrane Risk of Bias 2 tool.^16^ Overall risk of bias will be assessed in the following specific domains: the randomisation process (random sequence generation, allocation concealment); deviations from intended interventions, missing outcome data (low risk of bias if less than 10% missing data); measurement of the outcome (ie, measuring blood loss) and selection of the reported results. Each domain will be assigned a judgement according to published guidance. Where measures of quality are unclear, we will work proactively with relevant trialists to gain additional information.

### Integrity assessment

We will assess the trustworthiness of identified studies using a framework that incorporates key elements of the Cochrane Pregnancy and Childbirth Group screening tool^17^ and Trustworthiness in RAndomised Controlled Trials tool.^18^ Where measures of quality and integrity are unclear, we will work proactively with relevant trialists to gain additional information and refine the trustworthiness of included studies.

### Data analysis

Before embarking on data synthesis, collected data will be checked for accuracy and credibility. Characteristics of trials, and of patients recruited to the trials, will be summarised in descriptive tables. In the first instance, we will perform a suite of pairwise meta-analyses to understand the nature of the direct evidence. The primary analysis will combine the effect estimates across trials using the random-effect model due to anticipated heterogeneity. I^2^ statistics will be used to assess the statistical heterogeneity.^19^

### Outcomes and effect measures

We aim to assess the effects of interventions on outcomes that have been previously identified as important to stakeholders^20^,^22^; notably the main outcomes are

- Change in menstrual blood loss (assessed using pictorial blood loss assessment chart scores, the Alkaline-Haematin method or any other validated method).
- General and disease-specific quality of life (assessed using validated questionnaires).
- Treatment safety (specific to treatments under consideration) including complications, adverse events and serious adverse events.

Secondary outcomes of interest include post-treatment amenorrhoea, changes in haemoglobin, dysmenorrhoea, participants’ satisfaction and need for retreatment. The outcomes will be assessed at two time periods: (1) shorter (minimum of 3 months up to 6 months post-treatment) and (2) longer (any time after 6 months up to 12 months post treatment). If multiple time points are reported in the study, we will use the time points the closest to 6 and 12 months, respectively.

### Subgroup analyses

Where relevant, we will explore and perform subgroup analyses stratified by the following.

#### Trial level

Where treatments have been applied in different ways between included trials in any meta-analytic comparison (eg, variation in mode of delivery (oral, vaginal, injection)), then there may be variation in treatment effect observed. To explore these differences, providing sufficient data are available, we will group trials in subgroups defined by the trial-level characteristics. An effect estimate will be calculated for each trial group, and for all trials together, using the random-effect model (8) to give pooled estimates that represent the overall risk of an event on treatment compared with control. A test for interaction will be used to investigate if there are any substantial differences in the effect of treatment between these trial groups. We will appropriately take account of the inclusion of any multiarm randomised trials to avoid double-counting of patients.

#### Patient level

Providing sufficient data and power are available, we will investigate whether any treatment effect on the primary outcome is consistent across patient subgroups defined by age group/life stage (adolescent, pre-pregnancy, perimenopausal), prior treatment status (naïve, pretreated), desire for pregnancy and uterine anomaly (eg, fibroids, adenomyosis). Additional subgroups may emerge from consultations with relevant stakeholders. The interaction effect in each trial will be calculated from the ratio of the estimated treatment effects for each subgroup (eg, the pooled estimate of effect for treatment-naïve population, divided by that for pre-treated patients).

#### Treatment level

Providing sufficient data and power are available, we will investigate whether the treatment effect would vary by type of intervention (eg, hormonal, non-hormonal), type of composition (monotherapy vs combined treatment), and treatment dose.

The interaction effects will then be combined across trials using a fixed-effect meta-analysis.^23^ The method of Godolphin *et al*^24^ will be used to estimate pooled subgroup effect estimates, consistent with the pooled interaction, to avoid aggregation bias. Planned subgroups may be combined to achieve groups of a reasonable size. All p values will be two-sided.

### Sensitivity analyses

Random-effects meta-analyses, which estimate the average treatment effect across trials assuming a degree of between-trial heterogeneity, will be used. For our primary analysis, we will analyse the available data without imputation. However, if there is substantial missing data for an analysis of specific clinical interest, we may conduct sensitivity analyses where data are imputed using methods described in the *Cochrane Handbook*.^25^

Trial-specific sensitivity analyses will be conducted according to trial risk of bias (only studies at low risk of bias), integrity (only studies with high trustworthiness), publication date (before and after 2000) and size. We consider protocol publication in advance of the results to be an unsuitable criterion for sensitivity analyses because protocol publication only became widespread post 2000. Sensitivity analyses will be performed only for the primary outcomes.

### Network meta-analysis

Combining data from all trials in a network meta-analysis will enable us to assess the relative effectiveness of different treatment approaches on the primary outcome, using data from any direct, as well as from indirect comparisons to add strength to each of the treatment comparisons of interest. We will appropriately take account of patients from any multiarm randomised trials to ensure that they are not double-counted in the analysis.

This will provide the best opportunity to determine the relative effectiveness of treatments for which there are fewer trials/patients from which to draw conclusions.

The primary analysis will use a frequentist contrast-based approach implemented in multivariate meta-analysis models^26^ using the network suite of commands (Stata V.18.1) which assume consistency between ‘direct evidence’ (associations estimated in trials directly comparing the pair of interventions) and ‘indirect evidence’ (associations estimated through the network). The ‘net evidence’ from the network meta-analysis is a weighted average of the direct and indirect evidence. Inconsistency between direct and indirect evidence will be examined locally using symmetrical node-splitting^27^ and globally using a design-by-treatment interaction model.^28^

Borrowing of strength statistics will be calculated using the score decomposition method to illustrate the proportion of information for each net estimate that is due to indirect evidence. Treatment rankings will be calculated and are summarised according to the surface under the cumulative ranking curve value, which represents the rescaled mean ranking.^29^

### Assessing the certainty of evidence

We will use the Grading of Recommendations Assessment, Development and Evaluation (GRADE)^30^ or alternative approach like the certainty of evidence using the Confidence In Network Meta-Analysis approach^31^ to rate the certainty of the evidence for the overall effect across the included trials for the primary outcome. All analyses will be performed using Stata V.18 (StataCorp).

### Health economics evaluation

We will collect data on the costs and health benefits (quality-adjusted life-years (QALYs) wherever possible) associated with using each treatment option and their combinations in the NHS.

The choice for the most effective treatment for HMB should incorporate both the patient preference (eg, medical vs surgical), risk profile and potential cost implication in the healthcare sector. For example, undergoing a total hysterectomy would offer the maximum effectiveness in stopping HMB; however, this option will not be favoured by nulliparous younger women planning for pregnancy and would come with increased health costs in the short term.

The potential for cost saving that might be implied by some short-term medical treatment (eg, hormone-releasing intrauterine systems) might be offset by additional costs of repeated failed treatment, prolonged morbidity and adverse impact on quality-of-life measures in the long term.

A model-based economic analysis is ideally suited to collate the appropriate evidence from a range of sources and explore alternative scenarios and the uncertainty surrounding a range of possible results including subgroup analysis. Thus, if available data allow, the economic evaluation will be based on an outcome of cost per QALY and/or cost per morbidity-free survival post event.

We will include analyses based on a range of other outcomes, including treatment satisfaction, adverse events, days missed of work/school and other outcomes identified as important by women with evidence in the literature. The analysis will adopt a health service perspective. Once the clinical evidence has been synthesised to provide the relative effectiveness and side effects with the ranking of each treatment for the resolution of HMB, the relevant studies will be examined for their data on reported cost-related outcomes and resource use.

We will also search the wider literature for costs of event data. These data will be subjected to relevant quality criteria including GRADE and guidance set out by NICE Decision Support Unit and the Professional Society for Health Economics and Outcomes Research. Additional cost data will be available from other sources such as the National Schedule for Reference Costs and the British National Formulary. If necessary, primary cost and resource data will be collected from University College London Hospitals to complete any gaps in the information required for the modelling process. All costs will be expressed in 2024/2025 Great British pounds, other currencies will be converted using purchasing power parity and costs from different time periods will be adjusted using the NHS Cost Inflation Index.^32^

The evidence found in the systematic reviews will provide most of the parameters required to carry out the model-based economic evaluations of evaluated treatment options. Additional searches as part of a wider pragmatic review will be undertaken to help structure and populate the decision model. We will consult relevant stakeholders to identify the model questions. These questions may relate to preference-based health- related quality of life associated with HMB or analogous conditions; costs and duration associated with inpatient stay or side effects of the treatments or morbidity as a result of treatment for HMB. Information to answer these questions will be provided by focused searching of appropriate databases, including reference cost databases, statistical sources and other sources of relevant information.

We will develop a decision model using data from the systematic review considering the structure of women’s health services in the NHS. The model will be developed in consensus with relevant stakeholders (eg, clinicians and patient representatives) to reflect the current patient journey to access treatments. We will search the literature for evidence of existing model-based analyses for this clinical area and use these to inform our model structure as far as appropriate.

Given the relatively short-term impact of the intervention and treatment, the most appropriate model structure will be a simple decision tree, although we will explore other model structures including Markov models. We will leverage data from our evidence synthesis to construct the model based on the cost per QALY. However, experience from similar research suggests that appropriate data on QALY outcomes are likely to be limited. As such, we will not attempt to model a whole lifetime and will look for data and information that are related specifically to post-treatment outcomes with no specific time limit.

Where we are not able to find suitable parameters from the published sources to populate the model, we will make assumptions based on expert opinion and after the consultation with relevant stakeholders. A modelling framework is ideally suited to demonstrate and explore the importance of the inherent uncertainty. We will conduct and report the results of deterministic and probabilistic sensitivity analysis. An incremental approach will be adopted with a focus on additional costs and gain in benefits associated with a move away from the current treatment to resolve HMB to an alternative treatment. Net-monetary benefit (QALYS multiplied by a cost-effectiveness threshold) will be reported to allow comparisons across a range of treatment options. Costs and benefits will be discounted in line with NICE guidance.

The results of these economic analyses will be presented using cost-effectiveness acceptability curves to reflect sampling variation and uncertainties for a range of cost-effectiveness thresholds. Deterministic sensitivity analyses will be used to explore the robustness of these results to plausible variations in key assumptions and variations in the analytical methods used and consider the broader issue of the generalisability of the results.

### Discrete choice experiment

We aim to produce a health economic model that reflects the true health needs of women with HMB and incorporates their treatment preferences to inform health policy. Given the evolving health needs and priorities of women across different life stages, it is not clear if QALYs would sufficiently capture the outcomes of interest for women being treated for HMB. We will conduct a few focus groups (10–20 patient representatives) to determine the key outcomes of interest and treatment attributes for women with HMB. We will adopt representative sampling for a range of different ages, ethnicities and conditions.

We will then use the treatment rankings from our evidence synthesis and the patient input from the focus groups to construct treatment choice sets within a discrete choice experiment (DCE) that would help us model the treatment preferences of women with HMB. The DCE will be designed using Stata V.18.5. We aim to recruit 200–500 patient representatives (depending on the number of evaluated choices) who will be recruited over social media and key contracts of our lay collaborators (Katie’s Team and the Co-Production Collective). We will internally pilot the DCE to ensure the choice sets are understood by a lay audience and hold face and content validity. We will recruit participants to complete an online questionnaire built using Qualtrics software. They will be presented with an information sheet and a brief introductory video before they provide their consent and will continue to completing the questionnaire.

Participants will be asked to choose from treatment choice sets (each including scenario A or B) and select their preferred option using an online electronic survey software. This task will be repeated by varying the values for the key outcomes of interest. Participants’ responses will be analysed using methods of increasing complexity to determine which variables best explain treatment preferences. We will seek input from our patient representatives to assess the feasibility of including a cost variable that would allow us to calculate willingness to pay for different treatment outcomes. The results and final weightings of the DCE will then be incorporated to inform the design and decision of the planned model.

### Patient and public involvement

We developed a comprehensive PPI engagement strategy to ensure that we develop a good understanding of the perspectives of individuals living with HMB on treatment options available to alleviate this common health condition. The strategy will help us to ask the most relevant questions about treatments that are most important for individuals living with HMB are addressed, if possible, and where the gaps in research knowledge exist, so that these are appropriately communicated and acted on. Specifically, individuals living with HMB will contribute to decisions regarding outcome selection and identification of patient subgroups to be addressed, as well as in the interpretation of the results to ensure that the most effective, cost-effective and acceptable treatment options for HMB are incorporated into treatment guidelines and policy, to inform and improve practice.

Two PPI representatives will serve on the investigation team as equal co-investigators to inform the design and conduct of the study. We will recruit 2–3 women with lived experience of HMB and/or uterine fibroids to serve as members on the project oversight committee and advise on the project design, conduct and reporting. We will also establish a PPI group of 15–20 women with lived experience who will participate in two workshops during the project to inform choices for treatment comparisons, reported outcomes and treatment preferences. Two experienced PPI co-investigators will facilitate planned PPI activities to ensure wide, inclusive and diverse participation from various population subgroups of interest. PPI contributions will be renumerated in line with the National Institute for Health and Care Research (NIHR) recommendations.

## Ethics and dissemination

The planned evidence synthesis is exempt from NHS REC approval as a secondary research project. The project was approved by the UCL Institute for Women’s Health Low-Risk Research Ethics Committee (reference: 004_2023_24) for the planned patient involvement and qualitative research to support the planned analyses.

We also obtained approval from the UCL Research Ethics Committee (ID 16351/003) for the planned DCE and qualitative questionnaires. Participants will be provided with an information sheet and will consent to participating in the experiment online before moving on to complete the questionnaire. We will ensure all research activities are in line with the principles of General Data Protection Regulation (GDPR), Good Clinical Practice (GCP) and the Department of Health Research Framework.

The research question is inherently of high impact given the expressed institutional interest in this health topic from NICE, Royal College of Obstetricians and Gynaecologists, the government initiative for women’s health, several charities and service user groups. We aim to publish in high-impact peer-reviewed journals detailing the effectiveness, cost-effectiveness and side-effect profile of each HMB treatment option. We will disseminate the completed paper to the Department of Health, the NICE guideline development writing committee, the Scientific Advisory Committees of the relevant Royal Colleges and professional societies.

We will also present the findings at the key annual professional conferences and communicate them with NICE to inform further updates of their HMB guideline.^6^ We aim to produce a decision toolkit and evidence-based rank-o-gram to aid health professionals and patients in choosing the most appropriate treatment options.

## Discussion

Currently, there are several different treatment options offered in the NHS for managing heavy periods. However, access to these treatments varies across the UK depending on local care pathways and available resources. This often exposes affected women to inequitable care, reduces treatment satisfaction and prolongs their suffering from this condition.

A substantial body of evidence is emerging on several new treatments for HMB such as the use of oral gonadotropin-releasing hormone antagonists,^33^ novel endometrial ablation systems^10^ and selective progesterone receptor modulators.^34^ These treatments are particularly relevant to women with uterine fibroids and HMB, but are largely absent from existing reviews. While promising, substantial concerns exist regarding their safety (eg, liver injury following the use of ulipristal acetate),^35^ highlighting the need for comprehensive and up-to-date evidence synthesis to refine the recommendations in current clinical practice guidelines.

Current NHS treatment policy is largely governed by the NICE guideline^6^ which recommends the use of IUS as the first-line treatment option for women with no obvious pathology for HMB. The guideline, however, does not offer a clear ranking for clinical or cost-effectiveness when considering other alternative treatment options or women of different age groups.

Given this uncertainty, adoption of this guideline varies across NHS services. A UK survey showed significant variation in care provision, with only 38% of NHS hospitals providing a dedicated menstrual bleeding clinic and only 30% to have a local written protocol detailing the care pathway for women with HMB.^36^ Several studies highlighted the fragmented care and varied access to HMB treatments in the UK, leading to inequitable use of NHS resources.^37^,^39^ Women with more severe symptoms are more likely to seek surgical treatments for HMB (most vs least-severe quintile, 33.1% vs 56.0%; risk ratio (RR) 1.6, 95% CI 1.5 to 1.7); however, access to these services is influenced by several factors such as ethnicity, socio-economic deprivation and regional variations in care provision within the NHS.^36^

Identifying an evidence-based treatment initiation and escalation pathway is key to minimising the chronic adverse health impact of HMB and optimise patient access to desired treatments in a timely fashion. This will help strategically deliver and triage affected women to HMB services in the community while facilitating the provision of more specialised services (eg, minimal access surgery) across centres of excellence in secondary and tertiary NHS settings.

The main strength of our findings is the predicted high generalisability given the size of existing evidence and the potential to directly impact day-to-day patient care. The findings will help raise awareness of the available treatment options for HMB and reduce the stigma associated with seeking medical care for HMB. The planned comprehensive review of the literature will guide future research need and the provision of healthcare services across primary and secondary settings.

Several limitations will still apply. First, there are variations in the used outcome measurement tools particularly for the main of outcome of interest such as menstrual blood loss and quality of life which may contribute to increased heterogeneity and inconsistency in evidence networks. We aim to use standardised mean averages to adjust for these where relevant. Similarly, there is limited correlation between outcome threshold (eg, volume of menstrual blood loss) and clinical implications which may limit the generalisability of our findings across various population subgroups. Finally, using aggregate data will limit our ability to adjust for key effect modifiers such as age and ethnicity. To address this, we aim to apply a prospective meta-analysis within a collaborative approach to evidence synthesis to maximise the use of available data and evaluate potential interactions between various subgroups of interest.

## Supplementary material

10.1136/bmjopen-2024-085292 online supplemental file 1
