# Supplementary material for: Treatment options for women with heavy menstrual bleeding: a protocol for comprehensive systematic review, network meta-analyses and health economic assessment
Source: BMJ Open. 2025 Apr 22;15(4):e085292. doi: 10.1136/bmjopen-2024-085292 (PMC12015687; doi:10.1136/bmjopen-2024-085292)
Supplement: online supplemental file 1 [file bmjopen-15-4-s001.docx]

**Supplementary 1: Search strategy for planned systematic review, network meta-analyses, and health economic assessment on the treatment options for women with heavy menstrual bleeding.**

Master search strategy for Medline (via Ovid)

Item Term

1 menorrhagia/

2 menorrhag$.tw.

3 (menstrua$ adj5 (bleed$ or blood)).tw.

4 (heavy adj5 menstrua$).tw.

5 (dysfunctional adj5 uter$).tw.

6 hypermenorrh$.tw.

7 heavy menstrual bleeding.ab,ti.

8 heavy period$.ab,ti.

9 1 or 2 or 3 or 4 or 5 or 6 or 7 or 8

10 randomized controlled trial.pt.

11 controlled clinical trial.pt.

12 randomized.ab.

13 placebo.tw. 244794

14 clinical trials as topic.sh.

15 randomly.ab.

16 trial.ti.

17 crossover.tw.

18 cross-over.tw.

19 cross over.tw.

20 17 or 18 or 19

21 10 or 11 or 12 or 13 or 14 or 15 or 16 or 20

22 9 and 21

23 exp animals/ not humans.sh.

24 22 not 23

25 limit 24 to yr="2019 -Curren
