# Supplementary material for: Treatment options for women with heavy menstrual bleeding: a protocol for comprehensive systematic review, network meta-analyses and health economic assessment
Source: BMJ Open. 2025 Apr 22;15(4):e085292. doi: 10.1136/bmjopen-2024-085292 (PMC12015687; doi:10.1136/bmjopen-2024-085292)
Supplement: Supplementary file 2 [file bmjopen-2024-085292.reviewer_comments.pdf]

PEER REVIEW HISTORY

BMJ Open publishes all reviews undertaken for accepted manuscripts. Reviewers are asked to complete a checklist review form and are provided with free text boxes to elaborate on their assessment. These free text comments are reproduced below.

ARTICLE DETAILS

Title (Provisional)

Treatment options for women with heavy menstrual bleeding: A protocol for comprehensive systematic review, network meta-analyses, and health economic assessment.

Authors

Al Wattar, Bassel H; Rogozińska, Ewelina; Nicholson, Lily; Fisher, David; Bordea, Ekaterina; Hutchinson, Niccola Pascal; Moss, Ngawai; Hunter, Rachael; Khan , Khalid Saeed; Jurkovic, Davor; Tierney, Jayne; Vale, Claire L

VERSION 1 - REVIEW

**Reviewer**	**1**
**Name**	**Ragni, Margaret**
**Affiliation**	**University of Pittsburgh, Medicine, Hematology**
**Date**	**29-May-2024**
**COI**	**None.**

The manuscript by Wattar et al describes a planned comprehensive systematic review, network meta-analyses, and health economic assessment to assess treatment options for women with heavy menstrual bleeding (HMB).
Some comments:

1. *Etiology*: The most common disease group causing HMB is congenital bleeding disorders: von Willebrand disease (VWD), hemophilia, platelet disorders, and factor deficiency: do the authors plan to exclude this group? If so, this should be an explicit selection criteria. If the authors do include this group, they may want to consider a separate analysis, the inclusion of factor and other hemostatic agents in the cost-analysis, and consideration for a hematologist to advise on these disorders and their treatment on the analyses.

2. *Application*: Will results from the analyses of HMB (omitting bleeding disorders) have potential relevance and application for all women with HMB, in particular, for the women with HMB within the bleeding disorders community? Should they be the subject of this or a separate study?

3. *Introduction*: The most common cause of HMB is the congenital bleeding disorder, von Willebrand disease. Up to one third have iron deficiency anemia. Unless women with HMB are tested for VWD, the cause may remain obscure. Unless iron studies

are performed (Fe, TIBC, ferritin), iron deficiency will be missed and remain untreated. Testing for VWD should be considered in women with HMB, and considered (to include, to exclude) in the study.

4. *Primary Outcome*: PBAC, the primary outcome, is limited as it is a subjective measure. Are there other markers that could be assessed, e.g. cycle length, cycle severity, ferritin, days lost from work, ER visits, hospitalizations?
   What degree of *"change"* in PBAC will be considered significant, and by what measure: a fall in PBAC by some number of points, or a certain percentage change in PBAC, or a specific percentage reduction in PBAC from baseline? How will the authors model differing PBAC outcome assessments (number, absolute number, percentage reduction, etc.) across different studies? How will inherent differences in the various methods be handled?

5. *Secondary Outcomes*: Among the secondary outcomes, quality-of-life is an important variable to assess, but the correlation between PBAC and QoL in studies to date has been variable. Further, the type QoL tool used may differ between studies, some specific for HMB, "Ruta", vs. others nonspecific: how will differences in measures be handled?

6. *Therapeutic Interventions*: If more than one intervention is used to treat HMB, how will that be assessed: the current analysis includes single interventions. How will bias of a second treatment be handled in subgroup analyses, i.e. single vs several treatment groups? For example, if hormonal contraceptives are used daily at study onset, and tranexamic acid is allowed for intermittent use or for a few days of the menstrual cycle, how will these be analyzed? And how will attributable risk be assigned?

Reviewer 2

Name parry-smith, william

Affiliation Shrewsbury and Telford Hospital NHS Trust

Date 15-Sep-2024

COI chair of the Project Steering Committee for our NIHR funded research project titled: (Treatment options for women with heavy menstrual bleeding: A comprehensive systematic review, network meta-analysis, and health economic assessment).(NIHR153187)

This is a well considered protocol and will be conducted by an experienced team.

A few comments. The paper rightly points out that treatment options for HMB depend on where in her life course a women finds herself. I would like to see a more detailed analysis plan to capture and explore this key element. Figure one evidence synthesis frame work describes ' Women's satisfaction and compliance' I think this should be re-worded for great clarity. In the discussion- I am unclear how an evidence synthesis of treatment for HMB will lead to a rise in awareness and reduction in stigma associated with seeking care for HMB. A

strategy or intervention/ policy change is required for these two lofty aims rather than evidence synthesis per se?

Reviewer 3

Name Hu, Yanan

Affiliation Monash University, Department of Biostatistics Data Analytics Modelling and Health Economics

Date 12-Jan-2025

COI No competing interests to declare.

Thank you for offering me the opportunity to review this protocol paper. Please find my comments below.

1. Page 5, Subsection: Eligibility Criteria

Could the authors clarify whether they plan to include economic evaluation studies of RCTs that report on outcomes such as cost alone or quality of life alone? If so, please specify these details in the eligibility criteria.

2. Page 5, Subsection: Eligibility Criteria

Will the authors be including studies conducted outside of the UK? The protocol appears to be aimed at a UK readership, so it would be helpful to clarify whether studies from other countries will be considered.

3. Page 10, Line 5

The phrase “publication date (before and after 2000)” may require revision. If the authors intend to include studies from 2019 onwards, could the reference to 2000 be updated to reflect the correct starting point for inclusion?

4. Model-Based Economic Evaluation

How will the authors handle studies reporting economic evaluations in different currencies? If only UK-based studies are being considered, kindly disregard this comment. Otherwise, please provide more details on how currency differences will be addressed.

5. Page 15, Line 24

Could the authors clarify what is meant by "rank-o-gram"?

6. Discussion Section

It would be beneficial to include a limitations section in the discussion, acknowledging any potential constraints or weaknesses in the methodology.

7. Page 14, Subsection Discrete Choice Experiment

What tool or method will be used for patients to choose treatment options within the choice sets in the discrete choice experiment? Further details would help clarify the process.

VERSION 1 - AUTHOR RESPONSE

**Reviewers' comment**	**Authors' reply**
Reviewer: 1<br>Etiology: The most common disease group causing HMB is congenital bleeding disorders: von Willebrand disease (VWD), hemophilia, platelet disorders, and factor deficiency: do the authors plan to exclude this group? If so, this should be an explicit selection criteria. If the authors do include this group, they may want to consider a separate analysis, the inclusion of factor and other hemostatic agents in the cost-analysis, and consideration for a hematologist to advise on these disorders and their treatment on the analyses.	We thank the reviewer for their comment.<br><br>We do not plan to exclude this subgroup. As highlighted already in the methods section, we plan to conduct subgroup analyses per cause of HMB where possible.<br><br>No changes are needed to the manuscript.
Application: Will results from the analyses of HMB (omitting bleeding disorders) have potential relevance and application for all women with HMB, in particular, for the women with HMB within the bleeding disorders community? Should they be the subject of this or a separate study?	Thank you, as highlighted in the methods and dissemination sections, we plane to produce and report several subgroup meta-analyses to maximise the translation and generalisability of the finding to all women with HMB.<br><br>The relevance and application of our findings will vary depending on healthcare settings and key local regulatory factors; therefore, we cannot influence this.<br><br>No changes are needed to the manuscript.
Introduction: The most common cause of HMB is the congenital bleeding disorder, von Willebrand disease. Up to one third have iron deficiency anemia. Unless women with HMB are tested for VWD, the cause may remain obscure. Unless iron studies are performed (Fe, TIBC, ferritin), iron deficiency will be missed and remain untreated. Testing for VWD should be considered in women with HMB, and considered (to include, to exclude) in the study.	We thank the reviewer for their comment.<br><br>We followed the NICE guideline as the overarching framework for the planned analysis which reports that up to 50% of women with HMB have no underlying cause for this condition (https://cks.nice.org.uk/topics/menorrhagia-heavy-menstrual-bleeding/).<br><br>Testing to identify the underlying cause of HMB is within the diagnostic pathway and falls outside the scope of our study which is focused on treatment options.<br><br>No changes are needed to the manuscript.
Primary Outcome: PBAC, the primary outcome, is limited as it is a subjective measure. Are there other markers that could be assessed, e.g. cycle length, cycle severity, ferritin, days lost from work, ER visits, hospitalizations? What degree of "change" in PBAC will be considered	We agree with the reviewer that PBAC is not an accurate measurement tool. Identifying better outcome measurement tool is recommended for future research but is outside the remit of this protocol.

significant, and by what measure: a fall in PBAC by some number of points, or a certain percentage change in PBAC, or a specific percentage reduction in PBAC from baseline? How will the authors model differing PBAC outcome assessments (number, absolute number, percentage reduction, etc.) across different studies? How will inherent differences in the various methods be handled?	The choice of outcome has been prioritised by patient and public representatives with lived experience of HMB rather than the investigators and following on published core outcome set (https://www.comet-initiative.org/Studies/Details/789).<br><br>We will not enforce a specific threshold for clinical significance in PBAC, as reported in our methods, we will use standardised mean difference to maximise the use of available data and minimise variation across included studies. This is a standard Cochrane methodology when dealing with continuous outcomes which we have already referenced.<br><br>No changes are needed to the manuscript.
Secondary Outcomes: Among the secondary outcomes, quality-of-life is an important variable to assess, but the correlation between PBAC and QoL in studies to date has been variable. Further, the type QoL tool used may differ between studies, some specific for HMB, "Ruta", vs. others nonspecific: how will differences in measures be handled?	We agree that a degree of variation is expected when combining various QoL measurement tools.<br><br>As reported in our methods, we will use standardised mean difference to maximise the use of available data and minimise variation across included studies. This is a standard Cochrane methodology when dealing with continuous outcomes which we have already referenced.<br><br>No changes are needed to the manuscript.
Therapeutic Interventions: If more than one intervention is used to treat HMB, how will that be assessed: the current analysis includes single interventions. How will bias of a second treatment be handled in subgroup analyses, i.e. single vs several treatment groups? For example, if hormonal contraceptives are used daily at study onset, and tranexamic acid is allowed for intermittent use or for a few days of the menstrual cycle, how will these be analyzed? And how will attributable risk be assigned?	We already reported on this in the methods section:<br><br>Treatment level: Providing sufficient data and power are available, we will investigate whether the treatment effect would vary by type of intervention (e.g., hormonal, non-hormonal), type of composition (monotherapy vs. combined treatment), and treatment dose.
Reviewer: 2	
The paper rightly points out that treatment options for HMB depend on where in her life course a women finds herself. I would like to see a more detailed analysis plan to capture and explore this key element.	Thank you
Figure one evidence synthesis frame work describes ' Women's satisfaction and compliance' I think this should be re-worded for great clarity.	We thank the reviewer. We have amended this to Women's satisfaction and treatment adherence.
In the discussion- I am unclear how an evidence synthesis of treatment for HMB will lead to a rise in awareness and reduction in stigma associated with seeking care for HMB. A strategy or intervention/ policy	We agree with the reviewer that change in policy is needed.

change is required for these two lofty aims rather than evidence synthesis per se?	However, harmonising evidence and comparing all available treatment options will simplify the provision, counselling, and information provision to both health clinicians and women with HMB, thus yielding a positive change.<br><br>We have already highlighted this in our dissemination section.<br><br>No changes are needed to the manuscript.
Reviewer: 3<br>Page 5, Subsection: Eligibility Criteria<br>Could the authors clarify whether they plan to include economic evaluation studies of RCTs that report on outcomes such as cost alone or quality of life alone? If so, please specify these details in the eligibility criteria.	Thank you.<br>As reported, we will construct a de-novo health economic modelling that is fit for the NHS.<br><br>We will conduct a search of existing economic evaluation to fill gaps in our model where needed, but our objective is not to repurpose them.<br><br>We have clarified this as follow:<br><br>We will develop a decision model using data from the systematic review considering the structure of women's health services in the NHS. The model will be developed in consensus with relevant stakeholders (e.g., clinicians and patient representatives) to reflect the current patient journey to access treatments. We will search the literature for evidence of existing model-based analyses for this clinical area and use these to inform our model structure as far as appropriate.
Page 5, Subsection: Eligibility Criteria<br>Will the authors be including studies conducted outside of the UK? The protocol appears to be aimed at a UK readership, so it would be helpful to clarify whether studies from other countries will be considered.	Yes, as reported in our search strategy, no geographical search limitation will be employed.<br><br>No changes are needed to the manuscript.
Page 10, Line 5<br>The phrase "publication date (before and after 2000)" may require revision. If the authors intend to include studies from 2019 onwards, could the reference to 2000 be updated to reflect the correct starting point for inclusion?	To clarify, we aim to include all available studies with no date limitations. The proposed date of 1 January 2019 is only building upon existing Cochrane reviews that covered the previous period. We will use data from these Cochrane reviews to avoid duplication of efforts.<br><br>The proposed subgroup analysis of studies before and after 2000 is only to detect confidence in reported findings as protocol publication in advance of the results only became widespread post 2000.<br><br>We have already clearly reported on all these aspects in our methods section.

	No changes are needed to the manuscript.
Model-Based Economic Evaluation<br>How will the authors handle studies reporting economic evaluations in different currencies? If only UK-based studies are being considered, kindly disregard this comment. Otherwise, please provide more details on how currency differences will be addressed.	This not relevant to our planned economic analysis. As reported, we will develop a de-novo analysis specific to the NHS. We will only use clinical effectiveness data to calculate QALYs from the planned network meta-analysis. Cost estimated will be sought from NHS specific resources following the NICE framework to develop our health economic analysis.<br><br>We have already clearly reported on all these aspects in our methods section.<br><br>No changes are needed to the manuscript.
Page 15, Line 24<br>Could the authors clarify what is meant by "rank-o-gram"?	Rankogram is a method ranking available treatment options for their perceived effectiveness (e.g. from highest to lowest).<br><br>This is standard Cochrane methodology which we have reference in the method section.<br><br>No changes are needed to the manuscript.
Discussion Section<br>It would be beneficial to include a limitations section in the discussion, acknowledging any potential constraints or weaknesses in the methodology.	We agree and we have now included a limitation section as follow:<br><br>Several limitations will still apply. First, there are variations in the used outcome measurement tools particularly for main of outcome of interest such as menstrual blood loss and quality of life which may contribute to increased heterogeneity and inconsistency in evidence networks. We aim to use standardised mean averages to adjust for these where relevant. Similarly, there is limited correlation between outcome threshold (e.g. volume of menstrual blood loss) and clinical implications which may limit the generalisability of our findings across various population subgroups. Finally, using aggregate data will limit our ability to adjust for key effect modifiers such as age and ethnicity. To address this, we aim to apply a prospective meta-analysis within a collaborative approach to evidence synthesis to maximise the use of available data and evaluate potential interactions between various subgroups of interest.
Page 14, Subsection Discrete Choice Experiment<br>What tool or method will be used for patients to choose treatment options within the choice sets in the discrete choice experiment? Further details would help clarify the process.	We thank the reviewer for their comment. We have clarified that an electronic survey<br>tool will be used as follows:

	Participants will be asked to choose from treatment choice sets (each including scenario A or B) and select their preferred option using an online electronic survey software.

VERSION 2 - REVIEW

Reviewer 3

Name Hu, Yanan

Affiliation Monash University, Department of Biostatistics Data Analytics Modelling and Health Economics

Date 06-Mar-2025

COI

Thank you to the authors for their revisions. However, I still have some uncertainties regarding the following points:

1. Alignment of Cost Data Sources:

If, as stated, cost data will be sourced from NHS-specific resources, how does this align with the statement in your Health Economics Evaluation: "Once the clinical evidence has been synthesised to provide the relative effectiveness and side-effects with the ranking of each treatment for resolution of HMB, the relevant studies will be examined for their data on costs and resource use." and "We will also search the wider literature for costs of event data"

Specifically, I would like clarification on which studies are being referred to as "the relevant studies" in this sentence. If these are studies from the literature, it is possible that the cost data could originate from any country and be reported in any currency?

2. Date Limitation Clarification:

If, as stated, no date limitation was applied to the database search, then the sentence "Consequently, our literature **search** will cover a period from 1 January 2019" should be revised for clarity: "To eliminate duplication of efforts, we will only **extract** data for studies published from 1 January 2019."

It would be clearer to explicitly state that the **search** has no date limitation, but during the data synthesis phase, only studies published from 1 January 2019 will be included for data extraction. This distinction would help avoid any potential confusion.

3. There are two full stops at the end of the sentence "......to rate the certainty of the evidence for the overall effect of across the included trials for the primary outcome. . "

VERSION 2 - AUTHOR RESPONSE

Reviewers' comment	Authors' reply
If, as stated, cost data will be sourced from NHS-specific resources, how does this align with the statement in your Health Economics Evaluation: "Once the clinical evidence has been synthesised to provide the relative effectiveness and side-effects with the ranking of each treatment for resolution of HMB, the relevant studies will be examined for their data on costs and resource use." and "We will also search the wider literature for costs of event data"<br>Specifically, I would like clarification on which studies are being referred to as "the relevant studies" in this sentence. If these are studies from the literature, it is possible that the cost data could originate from any country and be reported in any currency?	We thank the reviewer for their comment.<br><br>As stated, this is an NIHR funded research with the aim of producing outputs specific for the UK National Health Service. We will use the cost monetary value using standard NHS costing in our health economic analysis.<br><br>The search and reporting on health economic data in included studies is aimed to highlight and contrast our analysis to previously published data specifically to measures used and reported outcomes for example QALYS.<br><br>For example, the cost of conducting a second-generation endometrial ablation is very different between the USA and the UK. So, using USA data is only helpful to make comparison.<br><br>As stated, All costs will be expressed in 2024/25 Great British Pounds (GBP), other currencies will be converted using Purchasing Power Parity (PPP) and costs from different time periods will be adjusted using the NHS Cost Inflation Index[32].<br><br>We have clarified this as follows:<br><br>We will include analyses based on a range of other outcomes including treatment satisfaction, adverse events, days missed of work/school and other outcomes identified as important by women with evidence in the literature. The analysis will adopt a health service perspective. Once the clinical evidence has been synthesised to provide the relative effectiveness and side-effects with the ranking of each treatment for resolution of HMB, the relevant studies will be examined for their data on reported cost related outcome and resource use.<br><br>We will also search the wider literature for costs of event data. These data will be subjected to relevant quality criteria including GRADE and guidance set out by NICE Decision Support Unit and The Professional Society for Health Economics and Outcomes Research (ISPOR). Additional cost data will be available from other sources such as the National Schedule for Reference Costs and the British National Formulary. If necessary, primary

	cost and resource data will be collected from University College London Hospitals to complete any gaps in the information required for the modelling process. All costs will be expressed in 2024/25 Great British Pounds (GBP), other currencies will be converted using Purchasing Power Parity (PPP) and costs from different time periods will be adjusted using the NHS Cost Inflation Index[32].
If, as stated, no date limitation was applied to the database search, then the sentence "Consequently, our literature **search** will cover a period from 1 January 2019" should be revised for clarity: "To eliminate duplication of efforts, we will only **extract** data for studies published from 1 January 2019."<br>It would be clearer to explicitly state that the **search** has no date limitation, but during the data synthesis phase, only studies published from 1 January 2019 will be included for data extraction. This distinction would help avoid any potential confusion.	Thank you, we have amended this accordingly as follows:<br><br>To avoid duplication of efforts, data extraction from previously published Cochrane reviews will be harvested. We will extract data from articles published after 1 January 2019 that met our inclusion criteria. Where additional data is required, we will review and extract data from all primary studies included in our review.
3. There are two full stops at the end of the sentence "......to rate the certainty of the evidence for the overall effect of across the included trials for the primary outcome. . "	Thank you, amended.
